# Water Use Patterns of Four Tropical Bamboo Species Assessed with Sap Flux Measurements

**DOI:** 10.3389/fpls.2015.01202

**Published:** 2016-01-07

**Authors:** Tingting Mei, Dongming Fang, Alexander Röll, Furong Niu, Hendrayanto, Dirk Hölscher

**Affiliations:** ^1^Tropical Silviculture and Forest Ecology, Georg-August-Universität GöttingenGöttingen, Germany; ^2^Department of Forest Management, Institut Pertanian BogorBogor, Indonesia

**Keywords:** calibration, environmental drivers, hysteresis, stem heat balance, thermal dissipation probes, trees, bamboos

## Abstract

Bamboos are grasses (Poaceae) that are widespread in tropical and subtropical regions. We aimed at exploring water use patterns of four tropical bamboo species (*Bambusa vulgaris*, *Dendrocalamus asper*, *Gigantochloa atroviolacea*, and *G. apus*) with sap flux measurement techniques. Our approach included three experimental steps: (1) a pot experiment with a comparison of thermal dissipation probes (TDPs), the stem heat balance (SHB) method and gravimetric readings using potted *B. vulgaris* culms, (2) an *in situ* calibration of TDPs with the SHB method for the four bamboo species, and (3) field monitoring of sap flux of the four bamboo species along with three tropical tree species (*Gmelina arborea*, *Shorea leprosula*, and *Hevea brasiliensis*) during a dry and a wet period. In the pot experiment, it was confirmed that the SHB method is well suited for bamboos but that TDPs need to be calibrated. *In situ*, species-specific parameters for such calibration formulas were derived. During field monitoring we found that some bamboo species reached high maximum sap flux densities. Across bamboo species, maximal sap flux density increased with decreasing culm diameter. In the diurnal course, sap flux densities in bamboos peaked much earlier than radiation and vapor pressure deficit (VPD), and also much earlier than sap flux densities in trees. There was a pronounced hysteresis between sap flux density and VPD in bamboos, which was less pronounced in trees. Three of the four bamboo species showed reduced sap flux densities at high VPD values during the dry period, which was associated with a decrease in soil moisture content. Possible roles of internal water storage, root pressure and stomatal sensitivity are discussed.

## Introduction

Bamboos (Poaceae, Bambuseae) are abundant in the natural vegetation of tropical and subtropical regions. They have been used by people for millennia and are still used as food and construction materials. In addition, a large variety of bamboo usages have been developed in recent decades, for example for pulp, paper, or clothing production (International Network for Bamboo and Rattan [INBAR], 2014). The increasing economic exploitation of bamboos goes along with a considerable expansion of bamboo plantations in some regions (Chen et al., 2009; Food Agriculture Organization [FAO], 2010), which may lead to changes in ecological processes such as water use patterns (Uchimura, 1994; Komatsu et al., 2010). Some bamboo stands were reported to evaporate more water than tree-dominated forests (Komatsu et al., 2010; Ichihashi et al., 2015), but studies focusing on water use patterns of bamboos are still rare thus far (Pereira and Hosegood, 1962; Dierick et al., 2010; Komatsu et al., 2010; Kume et al., 2010; Ichihashi et al., 2015).

Water use patterns of bamboos and trees differ in several aspects. In contrast to trees, bamboos are monocotyledonous species and lack secondary growth (Zimmermann and Tomlinson, 1972). Therefore, vascular conduits of bamboo xylem have to remain functional throughout the ontogeny of a bamboo culm. Bamboos consequently have great ability to avoid cavitation (Cochard et al., 1994; Cao et al., 2012; Petit et al., 2014); root pressure mechanisms may contribute to repairing embolized conduits at night (Cao et al., 2012). Such features and structural traits of bamboos may also lead to particular water use patterns.

In general, plant water use is driven by micrometeorological factors and can be limited by soil water availability (O’Brien et al., 2004; Bovard et al., 2005; Kume et al., 2007); it is regulated by stomata opening and closing (Jarvis, 1989) and can be influenced by internal water storage mechanisms (Waring and Running, 1978; Goldstein et al., 1998; Carrasco et al., 2014). Xylem sap flux reflects these multiple factors. For some tree species, for example, hysteresis in the diurnal sap flux response to radiation and vapor pressure deficit (VPD) of the air have been reported (Goldstein et al., 1998; O’Brien et al., 2004). Sap flux measurements thus appear suitable to study the water use patterns of bamboos as well as their controlling environmental factors.

Thermal dissipation probes (TDP) are widely used to measure sap flux density (*J*_s_) in trees (Granier, 1985). Several studies suggest calibrating the method before studying new species (Lu et al., 2004; Wullschleger et al., 2011; Vandegehuchte and Steppe, 2013). To our knowledge, only two studies have applied the TDP method on bamboos so far. Both reported an underestimation of bamboo sap flux compared to stem heat balance (SHB) and reference gravimetric measurements (GM) when the TDP method was not calibrated (Dierick et al., 2010; Kume et al., 2010). In contrast, the SHB method (Sakuratani, 1981) was suggested to be well suited for sap flux measurements on bamboos (Dierick et al., 2010). Bamboo culms are hollow; hence heat loss in the form of heat storage inside culms is marginal, so that steady thermal conditions as a main assumption of the method are met (Baker and Van Bavel, 1987).

The aim of this study was to analyze water use patterns of tropical bamboo species and particularly the response of *J*_s_ to the principal environmental drivers. First, we calibrated the SHB and the TDP method with reference GM in an experiment on potted culms of *Bambusa vulgaris*. We then measured *J*_s_ in the field in four bamboo species including *B. vulgaris* with both the TDP and SHB method, and calibrated the TDP method with the SHB method. Herein, three factors which may influence the quality of the calibration were tested: time step of the data, formula specificity and calibration formula type. After calibration of the TDP method, we applied it to monitor *J*_s_ in four bamboo and three tree species in a common garden in Bogor, Indonesia. Differences in the response of *J*_s_ to fluctuations in environmental conditions were assessed. The study intends to contribute to expanding the yet limited knowledge on the eco-hydrological functioning of bamboos.

## Materials and Methods

### Study Sites and Species Selection

The pot calibration experiment was conducted in Guangzhou, China (23°26′13″ N, 113°12′33″E, 13 m asl). The field calibration experiment and monitoring campaign were carried out in a common garden in Bogor, Indonesia (6°33′40″ S, 106°43′27″ E, 182 m asl). Average annual temperature in Bogor is 25.6°C and annual precipitation is 3978 mm. Relatively dry conditions with consecutive rainless days can occur between June and September. During this dry period, monthly precipitation is on average 40% lower than during the wet period (230 vs. 383 mm), and the number of consecutive dry days (rainfall < 1 mm) is twice that of the wet period (8 vs. 4 days, 1989–2008, Van Den Besselaar et al., 2014). During our study period (July 2012–January 2013), differences between dry and wet period were more pronounced, i.e., 155 vs. 489 mm monthly precipitation, 14 vs. 2 consecutive dry days, and 0.29 vs. 0.39 m^-3^ m^-3^ daily soil water content. In Bogor, four bamboo species (*B. vulgaris*, *Dendrocalamus asper*, *Gigantochloa atroviolacea*, *G. apus*) with five culms per species and three tree species (*Gmelina arborea*, *Shorea leprosula* and *Hevea brasiliensis*, **Table 1**) with five stems per species were selected and their *J*_s_ were monitored with the TDP method for 7 months.

**Table 1:** Structural characteristics of the studied bamboo and tree species (*n* = 5 per species; mean ± SD).

	Species	DBH (cm)	Bamboo culm wall thickness (cm)	Height (m)
Bamboo	*B. vulgaris*	7.0 ± 0.3	1.3 ± 0.1	17.9 ± 0.8
	*G. apus*	8.6 ± 0.4	1.2 ± 0.2	16.2 ± 2.7
	*D. asper*	11.9 ± 1.9	2.4 ± 0.2	21.1 ± 0.9
	*G. atroviolacea*	8.9 ± 0.6	1.6 ± 0.1	17.0 ± 1.0
Tree	*H. brasiliensis*	27.4 ± 2.3	-	25.2 ± 3.0
	*G. arborea*	26.3 ± 7.7	-	26.5 ± 2.3
	*S. leprosula*	20.7 ± 4.8	-	19.2 ± 2.5

*Culm wall thickness (derived from five culms per species) and culm height (derived from three cut culms per species) of the studied bamboos.*

### TDP Construction and Installation

To measure *J*_s_ in trees and bamboos, we used self-made TDP (1 and 2 cm length, respectively). In sensor design and construction, we followed Wang et al. (2012). Each TDP sensor was comprised of a heating (downstream) and a reference (upstream) probe made of steel hypodermic needles. The probes were placed 10 cm apart (vertically). For bamboos and trees, TDP installation depths in culms and stems were 1 and 2 cm, respectively. After installation, each TDP was supplied with a constant current of 120 mA; the respective power outputs of 1 and 2 cm length TDP were ∼0.1 and ∼0.2 W. TDP signals were sampled every 30 s and stored as 10-min averages for the pot calibration experiment and as 1-min averages for all other experiments by data loggers and multiplexers (CR1000, AM16/32, Campbell Scientific Inc., USA).

### Calibration of the TDP Method

#### Pot Calibration Experiment: TDP, SHB, and GM

**FIGURE 1:**
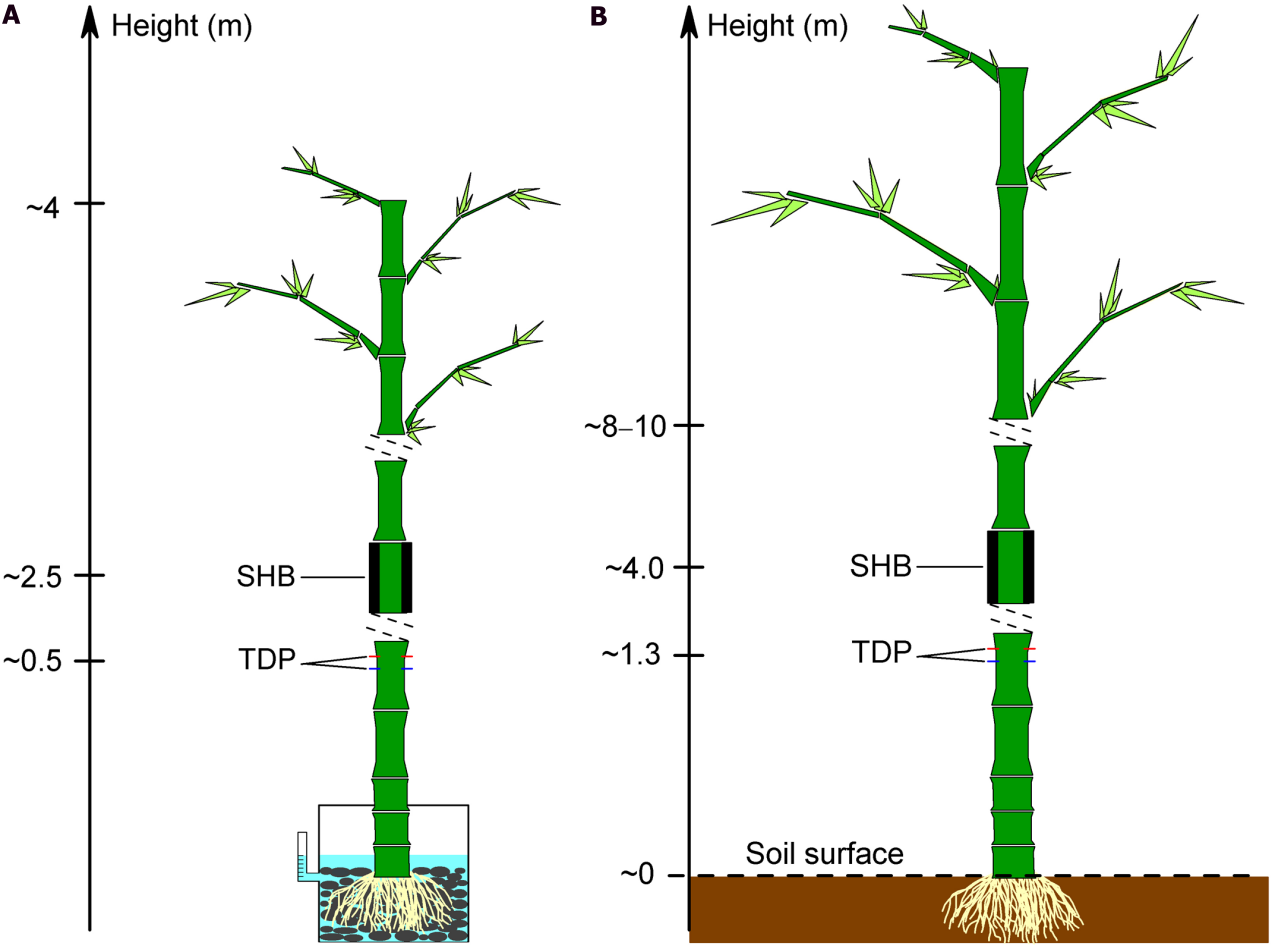
**Installation of thermal dissipation probe (TDP) and stem heat balance (SHB) sensors on bamboo culms for the calibration experiments on potted plants (A) and for field calibration (B)**.

Five culms of *B. vulgaris* (diameter 5.3–7.3 cm, height 2.2–3.2 m) with trimmed canopies were transplanted into plastic bags (diameter 30 cm, height 25 cm) 6 months before the calibration experiment. One day before the experiment, the five bamboos were transplanted into bigger plastic pots (diameter 50 cm, height 65 cm). The pots were filled with cobblestones and water and were then fully sealed with plastic cover and aluminum foil to prevent evaporation of water from the pots (**Figure 1A**). A scaled syringe tube was attached to each pot and connected into the pot through a U-type tube. At the beginning of the experiment, the water was added into the pot through the syringe tube to a fixed level (5 cm below the pot cover). Subsequently, water was added manually every 30 min to reach the pre-defined level. The weight of the added water was determined gravimetrically (GM). To measure *J*_s_, each bamboo culm was equipped with three pairs of 1 cm length TDP which were evenly installed circumferentially, about 15 cm above the plastic cover. To minimize potential measurement errors induced by circumferential variations of *J*_s_, the thermocouple wires of the three TDP were connected in parallel to get an average voltage signal for each bamboo culm (Lu et al., 2004). For a second *J*_s_ estimate, a SHB gage (SGB50 or SGA70, Dynagage Inc., USA) was installed about 1.5 m above the TDP. Both sensor types were protected by foil and the sensor signals were subsequently recorded as described in Section “TDP Construction and Installation.” For the comparison to reference GM, 10-min TDP and SHB derived values were aggregated to half-hourly values.

To assess the performance of TDP and SHB in the pot experiments, *J*_s_ derived from TDP and SHB (*J*_s_TDP_ and *J*_s_SHB_, respectively) on daily and 30-min scales were compared to GM derived *J*_s_ (*J*_s_GM_) with paired *t*-tests. Additionally, the slopes of the respective linear fits between *J*_s_TDP_, *J*_s_SHB_, and *J*_s_GM_ were tested for significant differences from one with the test of homogeneity of slopes. The same statistical analyses were applied again later when testing for significant differences between *J*_s_TDP_ and *J*_s_SHB_ in the field calibration experiments.

#### Field Calibration Experiment: TDP and SHB

Five culms per bamboo species (*B. vulgaris*, *D. asper*, *G. atroviolacea*, *G. apus*) were selected for TDP measurements (**Table 1**), three to four of which were additionally measured with SHB for a field calibration of the TDP method. TDP sensors were installed at 1.3 m height, and SHB gages (SGB50, SGA70, Dynagage Inc., USA) were installed about 2.5 m above the TDP. Simultaneous TDP-SHB measurements were conducted for a minimum of 5 days per culm (**Figure 1B**). Heat storage inside bamboo culms is assumed to be negligible, which was confirmed by installing thermocouple wires inside the measured segments of the respective bamboo culms to detect fluctuations in culm temperature (Dierick et al., 2010). The observed fluctuations were marginal, which meant stable thermal conditions as a requirement of the SHB method were met.

#### Parametrization for TDP Calibration

We derived cross-sectional water conductive areas (*A*_TDP_) from the culm wall thickness at the location of TDP sensor installation. In the pot calibration experiment, reference *J*_s_ were calculated by dividing water flow rates (g h^-1^, GM-derived) by *A*_TDP_. In the field calibration experiment, reference *J*_s_ were taken from the SHB measurements. The reference *J*_s_ could subsequently be used to calibrate *J*_s_TDP_. Nighttime sap flux values were excluded in both calibration experiments.

In the field calibration, three factors were considered for obtaining a TDP calibration formula from reference (SHB) measurements: time step of the data, formula specificity and calibration formula type. To examine effects of varying time steps, the formulas were built and tested on data at varying intervals (1-, 10-, 30-, and 60-min averages, respectively). The effects of formula specificity were examined by using common (i.e., all bamboo species pooled), species-specific and culm-specific formulas, respectively. Regarding the calibration formula type, two formulas were compared: one was non-linear (*J*_s_ = a*K*^b^) and generated by deriving new *a* and *b* parameters for the original Granier (1985) formula. The second was a linear formula (*J*_s_SHB_ = c × *J*_s_TDP_) which was calculated from the linear relationship between *J*_s_TDP_ and *J*_s_SHB_.

To obtain stable calibration formulas, pooled data sets were randomly split in half for calibration and independent validation, respectively (Niu et al., 2015). First, for each time step (1-, 10-, 30-, and 60-min, respectively), a data pool was built. Three culms of each bamboo species were randomly chosen, and for each, 3 days of data were randomly chosen from an initial common dataset. With these data pools, formula specificity was examined. For the common calibration, culms of all four species were selected for calibration. For species-specific and culm-specific calibration, only the data of the respective species or culms was selected. Next, the selected data was randomly split in half, for building the calibration formula and testing it, respectively. When testing the formula, the differences between *J*_s_SHB_ and calibrated *J*_s_TDP_ (*J*_s_TDP_cali_, abnormal distribution, *P* > 0.05) were examined with the Wilcoxon Signed-Rank Test (no significant differences at *P* > 0.05). The process of randomly building and testing the formula was iterated 10,000 times. Final calibration formula parameters were derived by averaging the parameters of those iterations which passed the Wilcoxon Signed-Rank Test (*P* > 0.05).

For an evaluation of the performance of the different formulas and the influence of the three factors (time scale, formula specificity and calibration formula type), differences in normalized Root-Mean-Square Errors (nRMSE) were assessed for each culm, species and formula factor, respectively. First, the RMSE for each day was derived with the *J*_s_SHB_ and *J*_s_TDP_cali_ values, and the nRMSE was calculated by normalizing the RMSE with the observed daily range of *J*_s-SHB_ (difference between maximum and minimum *J*_s_SHB_). Then, the nRMSE were analyzed regarding the three formula factors (data time scale, formula specificity and calibration formula type) by ANOVA (Analysis of variance). Additionally, for each day, *J*_s_TDP_cali_ with each formula type was tested for significant differences from *J*_s_SHB_ with the Wilcoxon Signed-Rank Test. The rates of passing the Wilcoxon Signed-Rank Test (*P* > 0.05 when no significant difference between TDP and SHB derived values) were assessed for each formula.

### Field Study

#### Monitoring Bamboo and Tree Sap Flux

Four calibrated bamboo species as well as three tree species (*G. arborea*, *S. leprosula*, and *H. brasiliensis*) were monitored with the TDP method for 7 months (July, 2012–January, 2013). Five bamboo culms and five tree trunks per species were selected for the measurements. On bamboos, three pairs of TDP (10 mm in length) were installed evenly around each culm at 1.3 m height and connected in parallel (see TDP Construction and Installation). On trees, two pairs of 20 mm TDP were installed in the trunk 1.3 m above the ground, in the North and South, respectively. *J*_s_ for the two sensors were separately derived with the original calibration formula (Granier, 1985) and subsequently averaged to obtain values for each tree. For bamboos, *J*_s_ derived with the original formula were calibrated with species-specific calibration parameters (from reference SHB field measurements) to obtain final *J*_s_ values.

#### Environmental Measurements and Analyses

A micrometeorological station was set up in an open area. It was about 100 and 600 m away from the closer measurement sites (*D. asper*, *G. arborea*, *G. atroviolacea*, *G. apus*, *S. leprosula*) and farthest sites (*B. vulgaris*, *H. brasiliensis*), respectively. Air temperature (Ta, °C) and air relative humidity (RH, %) were measured with a temperature and relative humidity probe (CS215, Campbell) installed in a radiation shield. VPD (kPa) was calculated from Ta and RH. Radiation (J m^-2^ s^-1^) was measured with a pyranometer (CS300, Campbell). Data were recorded with the previously described data loggers every minute.

In addition to the mentioned micrometeorological variables, soil moisture (SM, m^-3^ m^-3^) was measured with time domain reflectometry sensors (TDR, CS616, Campbell) at 0–20 cm depth. As the clump of *D. asper* and the stand of *G. arborea* were next to each other, one TDR was positioned between them to measure soil moisture. Likewise, one sensor was used for measurements of *G. atroviolacea* and *G. apus*. One TDR each were used for the remaining species (*S. leprosula*, *B. vulgaris*, *H. brasiliensis*). TDR measurements ran in parallel to the sap flux field campaign and data were recorded with the described data loggers every minute.

For the day-to-day analysis of influences of fluctuations in environmental conditions (VPD, radiation, SM) on *J*_s_ in the studied bamboo and tree species, daily accumulated *J*_s_ (kg cm^-2^ d^-1^) were normalized by setting the highest daily observation of each species to one and the lowest to zero. For a more isolated analysis of potentially limiting influences of soil moisture on *J*_s_, we focused on ‘dry period conditions’ with consecutive rainless days, which occurred between June and September in the study area. During this period, monthly precipitation was only 32% of monthly wet period precipitation (155 vs. 489 mm), and the number of consecutive dry days (rainfall < 1 mm) was seven times higher than during the wet period (14 vs. 2 days). Dry period conditions are also characterized by higher VPD (average daily VPD > 0.74 kPa on 92% of the days). 0.74 kPa was chosen as the threshold to distinguish between dry and wet period because it constituted the mean maximum (‘turning point’) in the fitted *J*_s_ response functions to VPD in three of the four studied bamboo species (except *D. asper*, see **Figure 4B**).

For the diurnal analysis of influences of fluctuations in environmental conditions on *J*_s_, time lags between *J*_s_ and micrometeorological drivers (radiation and VPD) were calculated as the time difference between the respective occurrences of maximal *J*_s_ (*J*_s_max_) and maximal radiation and VPD. *T*-tests were used to test time lags for significant differences from 0 min. 30-min *J*_s_ values (average values of three selected sunny days) of each species were plotted against radiation and VPD to examine occurrences of hysteresis. The respective areas of hysteresis were compared between bamboos and trees with *t*-tests.

All data analyses were performed with SAS 9.3 (SAS Institute Inc., Cary, NC, USA, 2013).

## Results

### Calibration of the TDP Method for Bamboos

#### Pot Calibration Experiment: TDP, SHB, and GM

**FIGURE 2:**
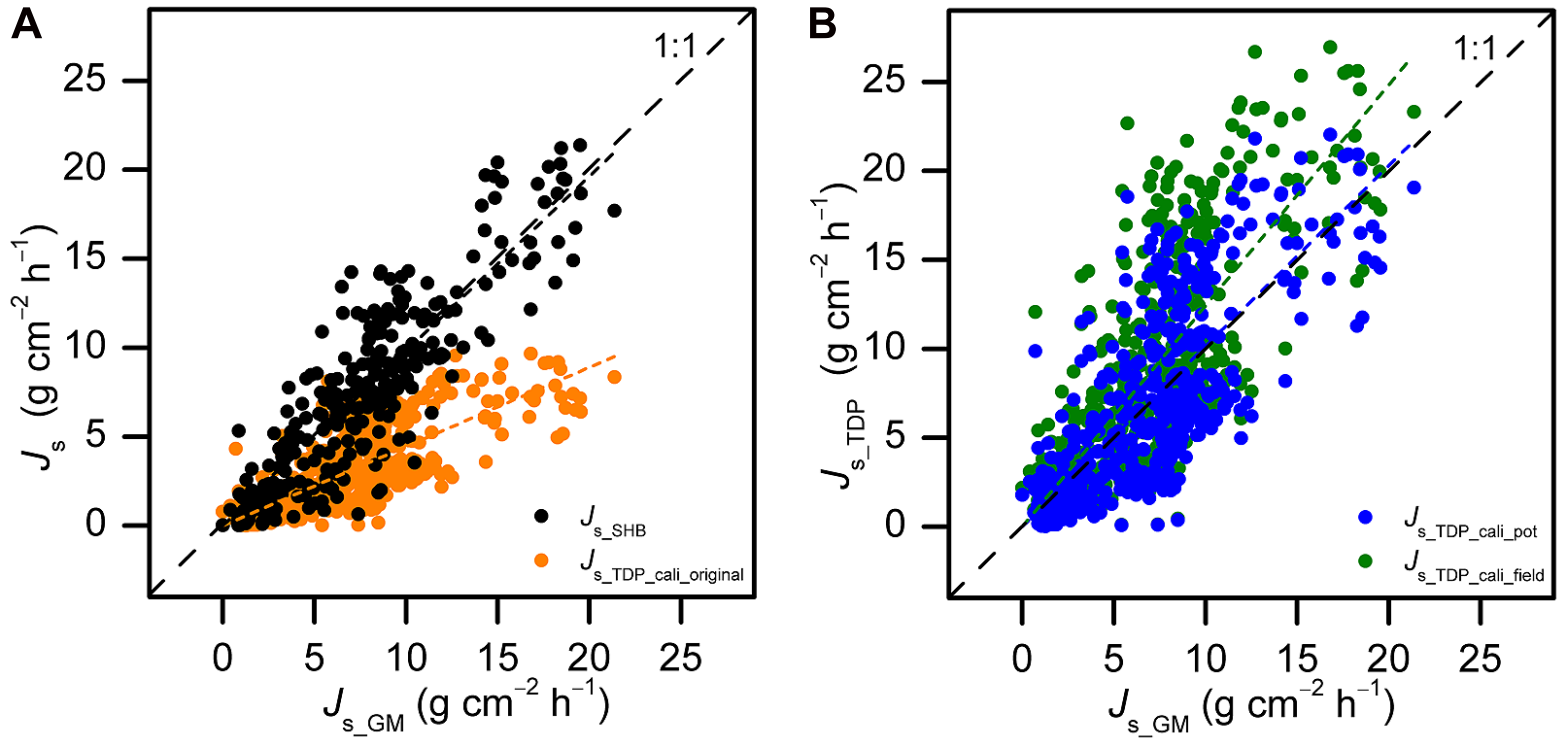
**Half-hourly sap flux density (*J*_s_) measured with thermal dissipation probes (TDP) and stem heat balance (SHB) sensors on five potted *Bambusa vulgaris* culms plotted against GM-derived reference sap flux densities (*J*_s_GM_) before (A; *J*_s_TDP_cali_original_: *Y* = 0.44X, *R*^2^ = 0.84, *P* < 0.01; *J*_s_SHB_: *Y* = 0.98X, *R*^2^ = 0.93, *P* < 0.01) and after (B; *J*_s_TDP_cali_field_: *Y* = 1.24X, *R*^2^ = 0.84, *P* < 0.01; *J*_s_TDP_cali_pot_: *Y* = 1.01X, *R*^2^ = 0.84, *P* < 0.01) species-specific calibration and field calibrations of the TDP method.** Pooled data from 2 to 5 days of simultaneous TDP, SHB, and gravimetric measurements (GM).

In the pot calibration experiment with *B. vulgaris*, SHB yielded similar absolute values of *J*_s_ as GM on daily and 30-min scales (*P* > 0.05). The slope of the linear fit between SHB and GM on the 30-min scale was 0.98 (*R*^2^ = 0.93, *P* < 0.01). It did not significantly differ from 1 (*P* > 0.05, **Figure 2A**). In contrast to this, TDP estimates, with the original parameters of the calibration formula (Granier, 1985), differed substantially from GM values at both the daily (60% underestimation of accumulated *J*_s_, *P* < 0.01) and 30-min scale (56% underestimation, *P* < 0.01). The slope of the linear fit between TDP and GM on the 30-min scale was 0.44 (*R*^2^ = 0.84, *P* < 0.01). It was significantly different from 1 (*P* < 0.01, **Figure 2A**).

After applying the TDP calibration parameter for *B. vulgaris* derived from the pot experiment (*c* = 2.28), the 30-min *J*_s_TDP_ were in line with those from GM. The slope was not significantly different from 1 (*P* > 0.05, **Figure 2B**). When applying the calibration parameters derived for *B. vulgaris* from the SHB field calibration experiment (*c* = 2.79), *J*_s_TDP_ was 19% higher than *J*_s-GM_ (*P* < 0.01, **Figure 2B**).

#### Field Calibration Experiment: TDP and SHB

Formula type and data time step had no significant influence on the performance of the calibration formula, but it mattered whether culm- or species-specific or a common calibration formula was used (Appendix Tables A1 and A2 Supplementary Material). Based on the nRMSE and the passing rate of the Wilcoxon test (*P* > 0.05) between calibrated *J*_s_TDP_ and *J*_s_SHB_, culm-specific formulas performed better than species-specific and common formulas. In our study, there was no statistically significant difference between the species-specific and the common calibration parameters (**Table 2**, *P* > 0.05). For two of the four studied bamboo species (*G. apus* and *B. vulgaris*), however, using species-specific formulas slightly improved the quality of predictions as compared to applying the common formula (*P* = 0.06 and 0.07, respectively, **Table 2**). These two bamboo species had lower nRMSE and higher passing rates than *D. asper* and *G. atroviolacea* (Appendix Table A2 in Supplementary Material). The linear calibration parameters of the four bamboo species were significantly different from each other (*P* < 0.01). The linear calibration parameters, the slopes of *J*_s_TDP_ vs. *J*_s_SHB_, were examined with the test of homogeneity of slopes and were found to differ significantly from each other (*t* > 0.01).

**Table 2:** Values of the parameter *c* of different bamboo calibrations (species-specific/common) for TDP sap flux estimates.

Formula specificity	Species	*c*	nRMSE		
			Species-specific formula	Common formula	*P*-value
Species	*B. vulgaris*	2.79 ± 0.13^a^	0.10	0.11	0.07
	*G. apus*	3.32 ± 0.08^b^	0.10	0.12	0.06
	*D. asper*	2.42 ± 0.06^c^	0.18	0.18	0.97
	*G. atroviolacea*	2.53 ± 0.11^d^	0.12	0.13	0.81
Common		2.74 ± 0.07^e^			

*Significant differences between species-specific and common c estimates (Tukey’s test, P < 0.01) are indicated by superscripted letters. P-values < 0.05 indicate significant differences between Normalized Root-Mean-Square Errors (nRMSE) of species-specific and common formula.*

Before calibration, *J*_s_TDP_ was on average 66 and 63% lower than SHB-derived reference values on the daily and 30-min scales, respectively (*P* < 0.01). This deviation was reduced to 10 and 8% underestimations (*P* < 0.01) when using species-specific calibration parameters (**Table 2**). On average, for 77 ± 6% of the days that were included in the analysis, the species-specific post-calibration 30-min *J*_s_TDP_ values were not significantly different from the respective reference *J*_s_SHB_ (Wilcoxon Signed-Rank test, *P* > 0.05).

### Field Study

#### Monitoring Bamboo and Tree Sap Flux

**FIGURE 3:**
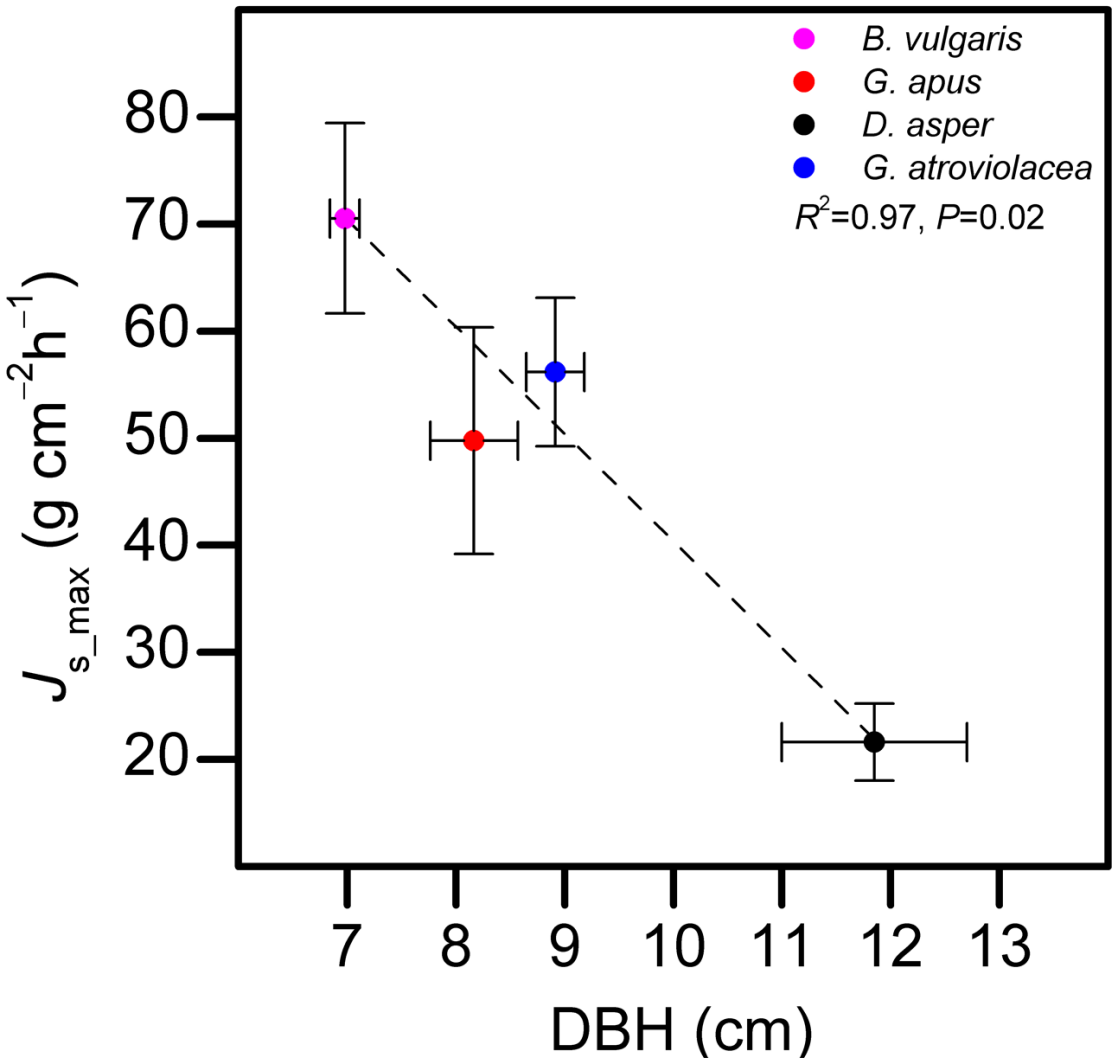
**Relationship between diameter at breast height (DBH) of bamboo culms and maximum observed sap flux density (*J*_s_max_) in four bamboo species.** Horizontal error bars indicate DBH standard errors, vertical bars standard errors of *J*_s_max_. Data of five culms pooled per species, average of the highest 10% of daily *J*_s_max_ values of each culm used for the analysis.

*J*_s_max_ in the studied bamboo species (averages from five individuals per species) were 70.5, 21.6, 49.7, and 56.2 g cm^-2^ h^-1^ for *B. vulgaris*, *D. asper*, *G. apus*, and *G. atroviolacea*, respectively. In trees, corresponding values were 17.7, 10.5, and 23.3 g cm^-2^ h^-1^ for *H. brasiliensis*, *G. arborea*, and *S. leprosula*, respectively. Across bamboo species, *J*_s_max_ decreased with increasing culm diameter (*R*^2^ = 0.97, *P* = 0.02, **Figure 3**).

#### Environmental Measurements and Analyses

**FIGURE 4:**
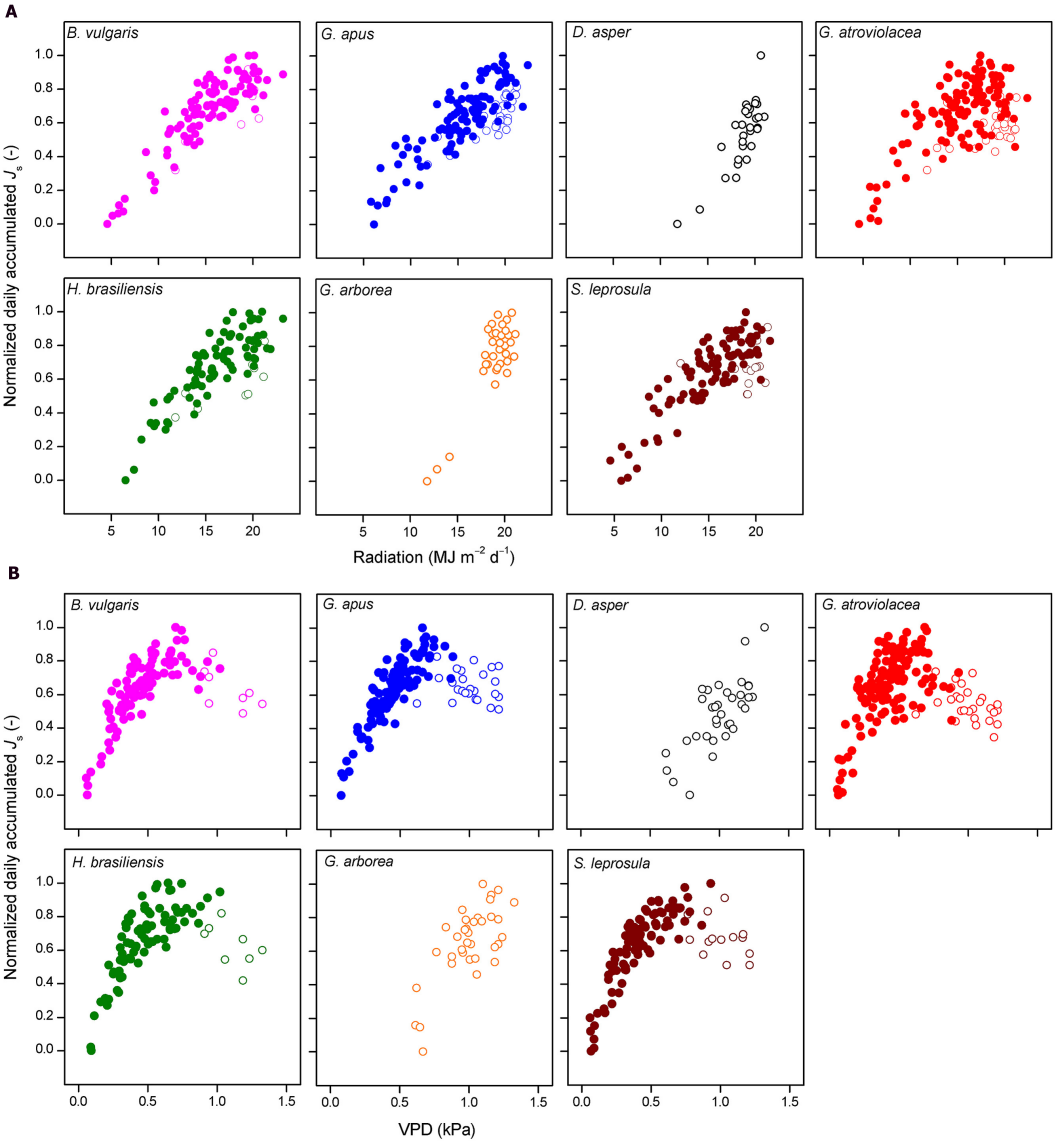
**Normalized daily accumulated sap flux density (*J*_s_) plotted against absolute values of (A) integrated daily radiation and (B) average daily vapor pressure deficit (VPD).** Daily values of four bamboo (upper row) and three tree species (lower row); data from 7 months of measurements (July 2012–January 2013) encompassing both wet (filled circles) and dry (open circles) periods (except for *Dendrocalamus asper* and *Gmelina arborea*, mainly dry period). Daily averages derived from measurements of five culms per species.

**FIGURE 5:**
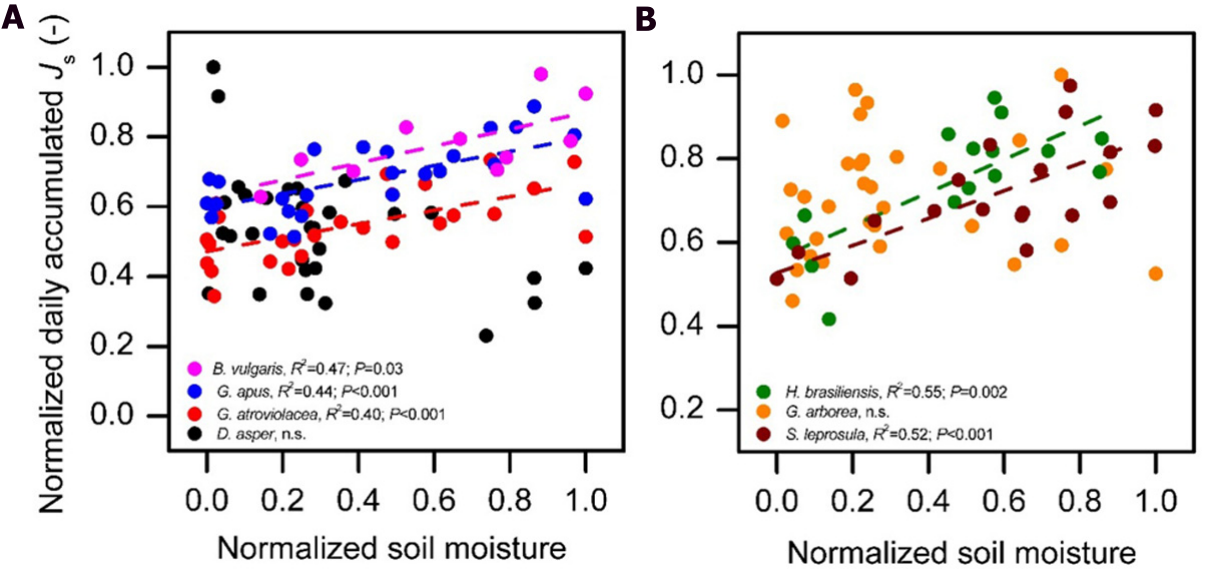
**Normalized daily accumulated sap flux density (*J*_s_) of four bamboo species (A) and three tree species (B) in the ‘dry period’ (characterized with mean daily VPD > 0.74 kPa) plotted against normalized mean daily soil moisture content (SM).** There was a significant linear relationship between *J*_s_ and SM (*P* < 0.05) for all species except *D. asper* and *G. arborea*. Normalized values do not reach 1.0 for all species in the figure as the normalization was performed by setting the maximum value of the full measurement period of each species (including wet period) to one, while the figure displays only values in dry period. Daily averages derived from measurements on five culms per species, data of at least 10 dry period days per species.

The normalized daily accumulated *J*_s_ of all studied species increased with increasing daily integrated radiation. This relationship did not fully hold up for accumulated *J*_s_ and average daily VPD. In several species, daily *J*_s_ increased with increasing VPD only to a certain VPD threshold (approximately 0.74 kPa, **Figure 4**); after this threshold, accumulated *J*_s_ decreased with further increasing VPD. Such conditions of high VPD were characteristic of the dry period. For days with VPD > 0.74 kPa, daily accumulated *J*_s_ of most studied species (except in *D. asper* and *G. arborea*) declined with decreasing soil moisture content (*R*^2^ = 0.39, 0.44, 0.4,0.52, and 0.55 for *B. vulgaris*, *G. apus*, *G. atroviolacea*, *S. leprosula*, and *H. brasiliensis*, respectively; *P* < 0.05, **Figures 5A,B**).

Diurnal peaks in *J*_s_ in the studied bamboo species occurred relatively early (on average at about 11 am), which was significantly earlier than the peaks of radiation and VPD (20–82 and 131–206 min, respectively). In the studied tree species, maximal hourly *J*_s_ values were observed after the peak of radiation (3–97 min), but still before (51–108 min) VPD peaked. All time lags were significantly different from 0 min (*P* < 0.01; **Table 3**), except for the time lag to radiation for the tree species *S. leprosula* (*P* > 0.05).

**Table 3:** Time lags between diurnal peaks of radiation and VPD and peaks of *J*_s_ in studied bamboos and trees.

Species	*N*	Time lag with radiation (min)	Time lag with VPD (min)
*B. vulgaris*	5	82 ± 62	171 ± 63
*D. asper*	5	41 ± 57	206 ± 57
*G. apus*	4	20 ± 61	131 ± 53
*G. atroviolacea*	5	64 ± 30	170 ± 35
**Bamboo_mean**	**19**	**51^A^**	**169^A^**
*H. brasiliensis*	5	-37 ± 12^a^	51 ± 9
*G. aborea*	5	-97 ± 87^b^	67 ± 87
*S. leprosula*	5	-3 ± 25^a^	108 ± 20
**Tree_mean**	**15**	**-46^B^**	**75^B^**

*Positive values indicate a peak of radiation/VPD after the peak of J_s_, negative values indicate a peak before J_s_; N, culms/trunks per species averaged (mean ± SD). Significant differences in bamboo/tree mean time lags are indicated by different superscripted letters (Tukey’s test, P < 0.01). Significant differences between species are indicated by capital letters (P < 0.01).*

**FIGURE 6:**
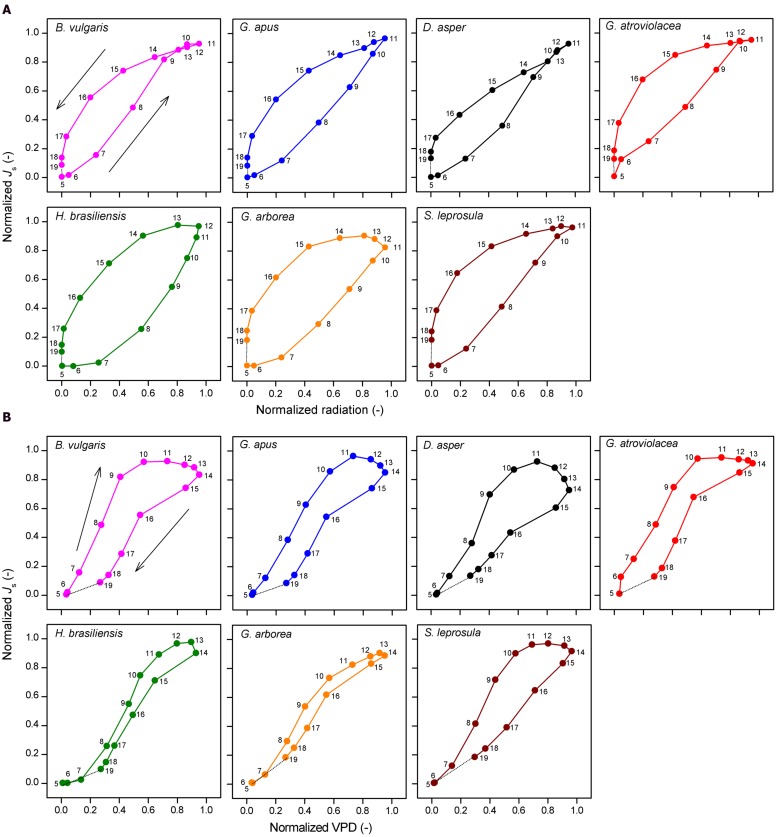
**Normalized hourly sap flux density (*J*_s_) plotted against (A) normalized hourly radiation and (B) VPD.** Data of four bamboo (upper row) and three tree species (lower row). Hourly averages derived from simultaneous measurements on five culms per species and by averaging the values of three sunny days to minimize influences of weather. The numbers in the sub-figures indicate the respective time of the day.

Diurnally, some of the studied species showed pronounced hysteresis of hourly *J*_s_ to radiation and VPD. Direction of rotation (i.e., order of observations) was counter-clockwise for radiation (**Figure 6A**) and clockwise for VPD (**Figure 6B**). The area of the hysteresis to VPD was on average 32% larger in bamboos than in trees, while the area of hysteresis to radiation was on average 50% smaller in bamboos (*P* < 0.01).

## Discussion

### Calibration Experiments

In the pot calibration experiment, SHB yielded similar results as reference GM measurements. Bamboos seem well suited for the SHB method (Dierick et al., 2010) due to their round shape and smooth and barkless surface, which allows for tight contact with the gages. Additionally, the hollow center and thin culm walls result in relatively low energy losses to heat storage so that the heat balance conditions required for the SHB method are met. ‘Zero sap flux’ conditions to obtain the heat conductivity of the sheath (*K*_sh_, Sakuratani, 1981) as a further requirement of the SHB method are difficult to determine *in situ* due to potential root pressure induced night time sap flux in bamboos (Cao et al., 2012); however, using *K*_sh_ derived from field conditions of very low night-time sap flux likely introduced only negligible errors into the calculation of daytime sap flux (Grime and Sinclair, 1999). As we observed very low sap flux over several hours during our experiments (e.g., about 1 g cm^-2^ h^-1^ during the pot experiment), our obtained *K*_sh_ were likely reliable.

In contrast to SHB, the TDP method was found to substantially underestimate *J*_s_ of bamboos in the pot and field calibration experiments. Underestimations by TDP were also reported in two other bamboo species: respective average underestimations of 13% for *B. blumeana* (Dierick et al., 2010) and 31% for *Phyllostachys pubescens* (Moso bamboo, Kume et al., 2010) were reported. Reasons for the observed underestimations could lie in the distinct hydraulic and physiological features of bamboos. Diurnal variations of stem water storage, for example, could affect the accuracy of TDP measurements (Vergeynst et al., 2014). Bamboos have approximately 50% parenchyma in culm walls (Dransfield and Widjaja, 1995), which potentially provides large water reservoirs. The depletion and refilling of the stem during the day and night, respectively, could cause diurnal fluctuations in culm thermal diffusivity. Higher water content during the night could lead to a lower maximum temperature difference (Δ*T*_max_) between heated and reference probe under “zero sap flux” conditions. Likewise, lower water content during the day could lead to higher observed Δ*T* values. As Δ*T*_max_/Δ*T* constitutes the basis for calculations of daytime *J*_s_, substantial underestimations of *J*_s_ could be introduced when using the original calibration parameters (Granier, 1985; Vergeynst et al., 2014). This hypothesis was assessed further by comparing the linear calibration parameters of *B. vulgaris* from the pot and the field calibration experiment (*c* = 2.28 and 2.79, respectively). In the pot experiment, the bamboos were always supplied with plenty of water, so that the variability of the culm water content was likely smaller than under field conditions. Effects of varying stem water content on Δ*T*_max_/Δ*T* are thus likely much smaller in the pot experiment, which may explain why pot and field calibration experiment yield different parameters for the linear calibration of the same species (*B. vulgaris*). Another potential factor for the divergence could be that the maximum observed *J*_s_ in the field (about 70 g cm^-2^ h^-1^) was much larger than in the pot experiment (about 20 g cm^-2^ h^-1^). Higher daytime sap flux (and thus transpiration) may cause a quicker depletion of the potential culm water storage, which consequently leads to a higher variability of culm water content between night and day.

We expected the calibration formula type (linear vs. non-linear) and data time step to have an impact on the performance of TDP predictions. However, both were not as important as the factor formula specificity. Even though species-specific calibration formulas generally did not perform significantly better than the common formula, species-specific formulas tended to show slightly better performance (**Table 1**) for two of the studied species (*G. apus* and *B. vulgaris*). Also, the calibration parameters were significantly different among the four studied bamboo species (**Table 2**). Confronting this insight with results from sap flux studies on other bamboo species (Dierick et al., 2010; Kume et al., 2010), differences among species become even more apparent. We thus used the derived species-specific formulas for further analysis. The observed differences among species may be indicative of highly heterogeneous wood anatomical properties among bamboo species. For example, size and shape of vascular bundles and parenchyma of 15 bamboo species were reported to be highly variable (Rúgolo de Agrasar and Rodríguez, 2003). For two further bamboo species (*Chusquea ramosissima* and *Merostachys claussenii*), it was suggested that differences in number of vascular bundles per unit area (1000 vs. 225 per cm^2^) and vessel length (∼1 m vs. 20 cm) could lead to differences in xylem hydraulic conductivity (Saha et al., 2009). Differences in wood anatomical properties may also lead to heterogeneous heat conductive properties, which potentially affects applicability and accuracy of sap flux measurements and particularly of the TDP method (Wullschleger et al., 2011).

In our study, culm-specific formulas performed better at predicting *J*_s_ than species-specific and common calibration formulas (Appendix Tables A1 and A2 in Supplementary Material). This result indicates heterogeneity in conductive properties among culms of the same species. Potential reasons could lie in the age and the ontogeny of individual culms. Even though we carefully selected culms of similar age (approximately 2 years old), the exact age of individual bamboo culms within a given clump is difficult to assess. As all monocot species, bamboos lack secondary growth (Zimmermann and Tomlinson, 1972), so culm diameters are not related to culm age. Additionally, over the ontogeny of a certain culm, events and processes such as conductive circuit failure (drought- or metabolism-related; Cochard et al., 1994; Liese and Weiner, 1996), lignification (Lin et al., 2002) or increasing hydraulic limitations with height (Renninger and Phillips, 2010; Cao et al., 2012) could result in overall reduced hydraulic conductivity and thus lower sap flux densities with increasing culm age. However, these processes remain difficult to assess from the outside of the culm; further studies linking the age and ontogeny of bamboos to (TDP-derived) sap flux and water use patterns are suggested.

### Water Use Patterns of Bamboos and Trees

Half-hourly *J*_s_max_ in the four studied bamboo species ranged from 21.6 to 70.5 g cm^-2^h^-1^ and were (on average) almost two times greater than in the studied tree species. The observed range for both bamboos and trees falls into the range of *J*_s_max_ values reported for tropical tree species in a variety of sap flux studies (Meinzer et al., 2001; O’Brien et al., 2004). For *D. asper*, the *J*_s_max_ (21.6 g cm^-2^ h^-1^) was similar to values reported for *B. blumeana* culms (25.7 g cm^-2^ h^-1^, Dierick et al., 2010) and Moso bamboos (approximately 20 g cm^-2^ h^-1^, Kume et al., 2010) of similar size. Our four studied bamboo species showed significant differences in *J*_s_max_, which were negatively correlated with species-specific differences in DBH (**Figure 3**). Consistent with this, in a study on 27 tropical tree species, the negative correlation between *J*_s_max_ and DBH was also observed (Meinzer et al., 2001). It was assumed to be related to a decline of the leaf area to sapwood area ratio with increasing DBH. This was also observed in a study on *Eucalyptus grandis* trees (Dye and Olbrich, 1993). In our study, we harvested leaves of three bamboo species (*B. vulgaris*, *D. asper*, and *G. apus*) and found that the leaf weight to sapwood area ratio was positively correlated with *J*_s_max_ (R^2^ = 0.45, *P* < 0.05). However, studies connecting such anatomical and eco-hydrological properties of bamboos are yet scarce (Saha et al., 2009).

On the day-to-day level, accumulated *J*_s_ of both the studied bamboo and tree species were significantly correlated with radiation and VPD (**Figure 4**). During the long wet period, accumulated *J*_s_ linearly increased with higher integrated radiation and average daily VPD. Likewise, linear relationships in the day-to-day behavior of *J*_s_ to micrometeorological drivers have been reported for some tropical bamboo and several dicot tree species (Dierick and Hölscher, 2009; Köhler et al., 2009). During the dry period characterized by higher radiation and VPD (13 and 100% higher, respectively) than during the wet period, however, the observed linear relationship to VPD did not hold. Higher average daily VPD (‘dry period conditions’) led to decreases in accumulated *J*_s_ of several studied species (**Figure 4B**). Similar decreases after a certain peak value have been reported for some previously studied tree species (Kubota et al., 2005; Jung et al., 2011), but in most species studied so far, higher average daily VPD leads to increases in accumulated *J*_s_ or water use (Wullschleger and Norby, 2001; Tang et al., 2006; Kume et al., 2007; Hernández-Santana et al., 2008; Peters et al., 2010; Horna et al., 2011). This was also reported for Moso bamboo (Komatsu et al., 2010). The observed decreasing accumulated *J*_s_ in bamboos under high VPD in our study were related to a reduction of soil moisture in the dry period (for three of the four bamboo and two of the three studied tree species). During the dry period, VPD was generally much higher than during the wet period. Soil moisture may become a limiting factor after several days without rainfall in the dry period. Accumulated *J*_s_ decreased strongly and linearly with decreasing soil moisture under ‘dry period conditions’ (i.e., VPD > 0.74 kPa) for all studied bamboo (except *D. asper*) and tree species (except *G. arborea*, **Figure 5**). Similarly, in a throughfall reduction experiment in Indonesia, declines of monthly *J*_s_ of Cacao and *Gliricidia sepium* were found to linearly correlate with reduced soil moisture (Köhler et al., 2010). Such sensitivity of daily *J*_s_ to fluctuating soil moisture may be related to a relatively shallow rooting depth (Kume et al., 2007).

Regarding the diurnal course of *J*_s_, the studied bamboo species showed earlier peaks than radiation and VPD, and also earlier than the respective peaks of the studied tree species. In contrast to this, previous studies on tropical trees reported rather small time-lags between peaks of *J*_s_ and radiation and VPD, respectively (Dierick and Hölscher, 2009; Köhler et al., 2009; Horna et al., 2011). Pre-noon peaks of *J*_s_ have only been described for few species thus far, for example, *Acer rubrum* (Johnson et al., 2011) and oil palms (Niu et al., 2015). The early diurnal peaks of *J*_s_ result in substantial hysteresis of *J*_s_ particularly to VPD. For another monocot species, oil palm, it has been suggested that such pre-noon peaks of *J*_s_ and the resulting large hysteresis to VPD could be indicative of internal trunk water storage and/or root pressure mechanisms (Niu et al., 2015; Röll et al., 2015). Early peaks of *J*_s_ could be due to a pre-noon contribution of internal water storage to bamboo transpiration. Likewise, the decoupling of hourly *J*_s_ particularly from VPD in the afternoon, i.e., the drop in bamboo *J*_s_ (after an early peak) despite further rising VPD, could be connected to the reduced water availability for leaves after the depletion of internal water storage at a certain time of the day. The depletion of stored stem water may be compensated for during the night by root pressure mechanisms (Cao et al., 2012; Yang et al., 2012). Other potential reasons for the diurnally relatively early decline of bamboo *J*_s_ and the consequent decoupling of the sap flux response from micrometeorological drivers could be a decline in leaf hydraulic conductance in the afternoon hours, which could contribute to prevent stem water potential loss and subsequent xylem cavitation (Saha et al., 2009; Yang et al., 2012).

## Conclusion

Adjusting and applying the TDP method for sap flux measurements on four bamboo species pointed to substantial differences in water use patterns between the studied bamboos and three tree species studied. Bamboos had higher *J*_s_, and respective hourly maxima were reached earlier in the day than in tree species. This resulted in strong diurnal hysteresis, particularly to VPD, and in significant time lags between the peaks of *J*_s_ in bamboos and the respective peaks of radiation and VPD. Both may point to a strong contribution of internal water storage mechanisms to bamboo transpiration. We found substantial differences in the day-to-day *J*_s_ response of most studied bamboo and tree species to fluctuations in environmental conditions between the dry and the wet period. Reduced *J*_s_ under conditions of high VPD in the dry period could largely be explained by limiting soil moisture content. The regulation of bamboo water use thus seems to involve mechanisms at the leaf-, culm-, and root- level. However, these mechanisms yet remain to be inter-connected convincingly.

## Conflict of Interest Statement

The authors declare that the research was conducted in the absence of any commercial or financial relationships that could be construed as a potential conflict of interest.
