# Supplementary material for: Water Use Patterns of Four Tropical Bamboo Species Assessed with Sap Flux Measurements
Source: Front Plant Sci. 2016 Jan 7;6:1202. doi: 10.3389/fpls.2015.01202 (PMC4703849; doi:10.3389/fpls.2015.01202)
Supplement: Supplementary file 1 [file Table_1.DOC]

**Appendix Table1. The influence of the three factors formula type, time step and formula specificity on the performance of the linear calibration model. Results of multi-ANOVA of the three factors against normalized Root-Mean-Square Error (nRMSE, calculated by normalizing the RMSE with the observed range of sap flux densities from stem heat balance (SHB) measurements; RMSE derived from SHB measurements vs. model-predicted values of each day). Data of 63 days were used. P<0.01 indicates significant difference.**

| Source | DF | Type III SS | Mean Square | F Value | P |
| --- | --- | --- | --- | --- | --- |
| Formula type | 1 | 0.0046 | 0.0046 | 0.68 | 0.4093 |
| Time step | 3 | 0.0046 | 0.0015 | 0.23 | 0.8777 |
| Formula specificity | 2 | 7.7675 | 3.8838 | 577.54 | <.0001 |
