# Supplementary material for: Water Use Patterns of Four Tropical Bamboo Species Assessed with Sap Flux Measurements
Source: Front Plant Sci. 2016 Jan 7;6:1202. doi: 10.3389/fpls.2015.01202 (PMC4703849; doi:10.3389/fpls.2015.01202)
Supplement: Supplementary file 2 [file Table_2.DOC]

**Appendix** Table2. Performance of culm-specific vs. species-specific vs. common linear formulas for a simple linear field calibration of the thermal dissipation probe (TDP) method with the stem heat balance (SHB) method on four bamboo species Normalized Root-Mean-Square Errors (nRMSE) and passing rates of the Wilcoxon Signed-Rank test for each species and formula specificity type. Superscripted letters indicate significant differences between nRMSEs and passing rates, respectively, within each species (Tukey's test, P<0.05).

| **Species** | **Formula**  **specificity** | **nRMSE** | **Passing rate (%)** |
| --- | --- | --- | --- |
| *B. vulgaris* | common | 0.11^a^ | 83^a^ |
|  | culm | 0.04^b^ | 94^b^ |
|  | species | 0.10^a^ | 84^a^ |
| *G. apus* | common | 0.12^a^ | 77^a^ |
|  | culm | 0.06^b^ | 90^b^ |
|  | species | 0.10^a^ | 81^a^ |
| *D. asper* | common | 0.18^a^ | 74^a^ |
|  | culm | 0.04^b^ | 94^b^ |
|  | species | 0.18^a^ | 70^a^ |
| *G. atroviolacea* | common | 0.13^a^ | 74^a^ |
|  | culm | 0.06^b^ | 89^b^ |
|  | species | 0.12^a^ | 74^a^ |
